# Circular RNA circVEGFC accelerates high glucose-induced vascular endothelial cells apoptosis through miR-338-3p/HIF-1α/VEGFA axis

**DOI:** 10.18632/aging.103478

**Published:** 2020-07-17

**Authors:** Hua Wei, Cong Cao, Xiaojuan Wei, Minglv Meng, Biaoliang Wu, Lianxin Meng, Xi Wei, Shixing Gu, Hongmian Li

**Affiliations:** 1 Department of Endocrinology, The Affiliated Hospital of Youjiang Medical University for Nationalities, Baise 533022, Guangxi, China; 2 Urology Care Unit, The Affiliated Hospital of Youjiang Medical University for Nationalities, Baise 533022, Guangxi, China; 3 Medical Statistics Office, Youjiang Medical University for Nationalites, Baise 533022, Guangxi, China; 4 Department of Plastic and Aesthetic Surgery, The Fifth Affiliated Hospital of Guangxi Medical University, Nanning 530021, Guangxi, China

**Keywords:** circular RNA, circVEGFC, vascular endothelial cells, high glucose, VEGF

## Abstract

More and more findings illustrate the critical roles of circular RNA (circRNA) in diabetes mellitus (DM) and its complications. A major pathological characteristic for DM is the apoptosis of endothelial cells (ECs) induced by high glucose (HG), however, the function of circRNA in the ECs’ phenotypes is still elusive. Here, this study identified an up-regulated circRNA (circVEGFC) in the HG-induced human umbilical vein endothelial cells (HUVECs). Functionally, knockdown of circVEGFC alleviated the apoptosis and recovered the proliferation in HUVECs induced by HG administration. Mechanistically, circVEGFC functioned as the sponge of miR-338-3p, and miR-338-3p was found to target the 3’-Untranslated Regions (3’-UTR) of hypoxia inducible factor 1 alpha (HIF-1α). HIF-1α, a critical transcription factor in DM, could activate the transcription of vascular endothelial growth factor A (VEGFA) and promote its protein product. In conclusion, these findings reveal the promotion of circVEGFC/miR-338-3p/HIF-1α/VEGFA axis in the HG-induced ECs’ apoptosis, providing a potential treatment strategy for ECs’ damage in DM.

## INTRODUCTION

Diabetes mellitus (DM) is a group of metabolic diseases characterized by hyperglycemia [1, 2]. In the pathological condition of DM, hyperglycemia results in chronic damage and dysfunction of multiple organs, especially heart, brain, blood vessels, eyes, and kidneys [3]. The pathologic basis of multiple organ damage is principally attributed to dysfunction of vascular endothelium [4, 5]. From the perspective of development, high glucose (HG) induced endothelial dysfunction, including vascular endothelial cell (ECs) and smooth muscle cell, is considered to be critical contributor for diabetic atherosclerosis and other diabetic vascular complications.

Circular RNAs (circRNAs), a covalently-bonded RNA transcript from back-splicing of linear RNA ad derived from precursor mRNA, are considered as the novel regulator in human disease [6]. Because circular RNAs are back-splicing products and covalently closed transcripts, they are more stable and resistant to decay machineries [7]. Emerging evidences demonstrated that circRNA participate in the pathological initiation and progression of vascular ECs and smooth muscle cells. For example, a classical circRNA, circHIPK3, could inhibit the HG-induced endothelial cell apoptosis through sponging miR-124 [8]. In oxLDL induced HUVECs, hsa_circ_0003575 is validated to be significantly up-regulated to regulate proliferation and apoptosis and bioinformatics predicts the potential circRNA-miRNA-mRNA network in the HUVECs [9].

In this research, circular RNA microarray analysis demonstrated the circRNA prolife in the HG-induced and NG-induced HUVECs. Here, circVEGFC (hsa_circ_0071465, splicing length 441 bp) was derived from the VEGFC gene exon-6 and exon-5. In HG-induced HUVECs, circVEGFC knockdown could reduce the apoptosis and promote proliferation through miR-338-3p/HIF-1Α/VEGFA axis. In conclusion, these findings reveal the promotion of circVEGFC/miR-338-3p/HIF-1α/VEGFA axis in the HG-induced ECs’ apoptosis, providing a potential treatment strategy for ECs’ damage in DM.

## RESULTS

### Circular RNA microarray revealed the novel circVEGFC in HG-induced HUVECs

**Figure 1:**
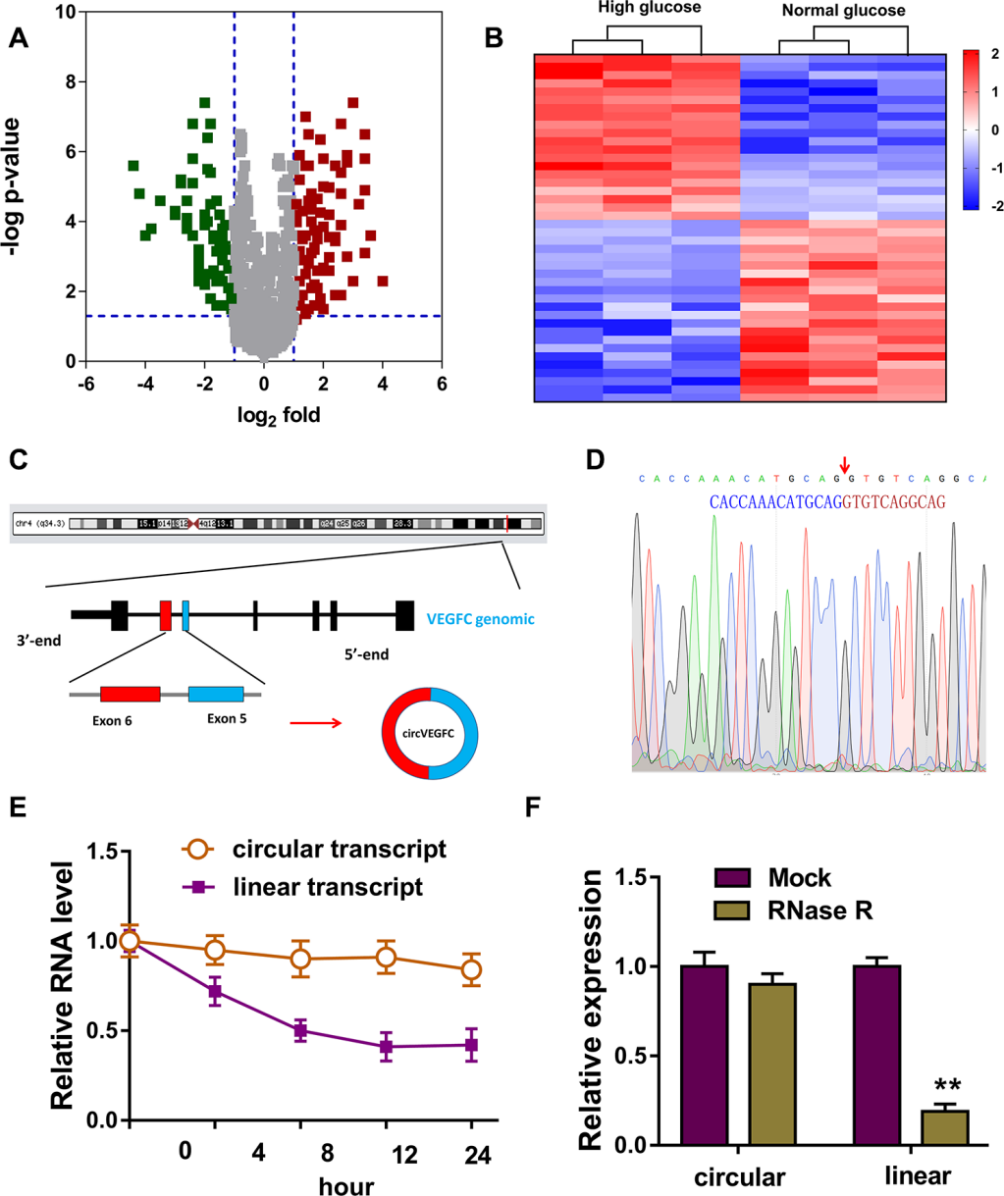
**Circular RNA microarray revealed the novel circVEGFC in HG-induced HUVECs.** (**A**) Volcano plot revealed the up-regulated circRNAs (red) and down-regulated circRNAs (green) in the microarray analysis. (**B**) Heat map demonstrated the dysregulated circRNAs in the microarray sequencing. (**C**) Schematic diagram illustrated the biogenesis of circVEGFC from the exon-6 and exon-5 of VEGFC gene locus. (**D**) The junction sites of circVEGFC was validated using the Sanger sequencing. (**E**) RNA stability assay revealed the circular or linear transcript form when treated with transcription inhibitor actinomycin D. (**F**) RT-PCR revealed the linear or circular transcript form of VEGFC when treated with RNase R. **P < 0.01.

To unveil the expression profile of circRNA in the HG or NG induced HUVECs, circRNA microarray analysis was performed. Volcano plot revealed the up-regulated circRNAs (red) and down-regulated circRNAs (green) in the microarray analysis (Figure 1A). Heat map demonstrated the dysregulated circRNAs in the microarray sequencing (Figure 1B). Schematic diagram illustrated the biogenesis of circVEGFC. circVEGFC was generated from the exon-6 and exon-5 of VEGFC gene locus (Figure 1C). The junction sites of circVEGFC was validated using the Sanger sequencing (Figure 1D). RNA stability assay revealed that the circular transcript form of circVEGFC was much more stable than the its linear transcript when treated with actinomycin D, a transcription inhibitor (Figure 1E). Moreover, RT-PCR revealed that linear transcript form of VEGFC was significantly decreased when treated with RNase R, however, the circular transcript form of circVEGFC was stable (Figure 1F). Overall, this evidence concluded that the novel circVEGFC was up-regulated in HG-induced HUVECs.

### circVEGFC knockdown alleviated the apoptosis and proliferation inhibition induced by HG

**Figure 2:**
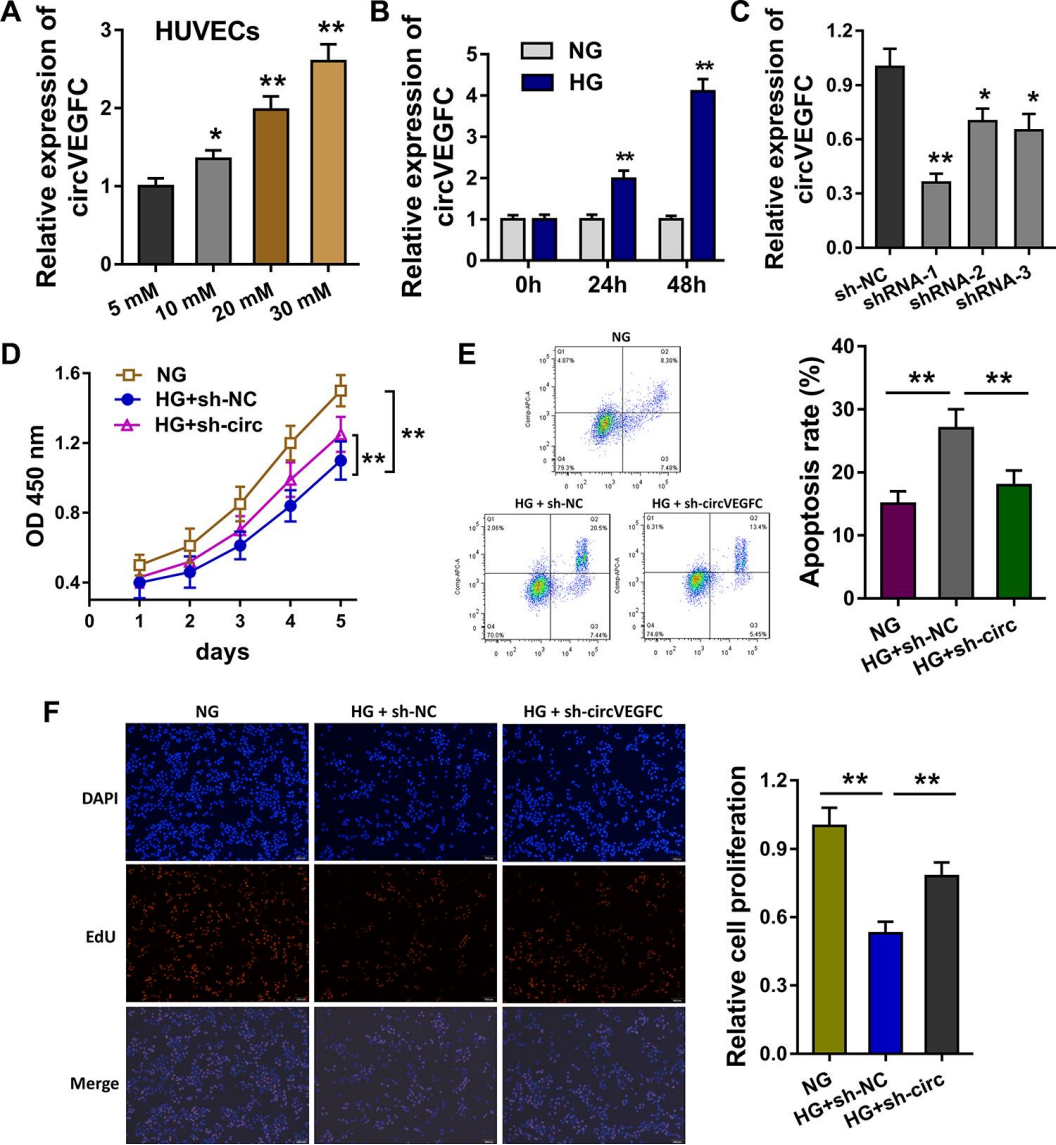
**circVEGFC knockdown alleviated the apoptosis and proliferation inhibition induced by HG.** (**A**) RT-qPCR showed the level of circVEGFC in the HUVEC cells treated with HG concentration gradient. (**B**) The expression level of circVEGFC with different time. (**C**) Short hairpin RNA (shRNA) was synthesized to knock down the circVEGFC expression. (**D**) CCK-8 assay indicated the proliferative ability of HUVECs with normal glucose (NG)/HG administration or circVEGFC knockdown transfection. (**E**) Flow cytometry apoptosis unveiled the apoptosis of HUVECs with NG/HG administration or circVEGFC knockdown. (**F**) Ethynyl-2-deoxyuridine (EdU) demonstrated the proliferation of HUVECs transfected with circVEGFC knockdown or control. *P < 0.05, **P < 0.01.

In the HUVEC cells, the HG administration could induce the expression level of circVEGFC with concentration gradient effect upon HG dosage (Figure 2A). Moreover, the level of circVEGFC was increased as time went on (0h, 24h, 48h) (Figure 2B). Short hairpin RNA (shRNA) was synthesized to knock down the circVEGFC expression (Figure 2C). CCK-8 assay indicated that HG administration decreased the proliferative ability, while circVEGFC knockdown recovered the proliferation (Figure 2D). Flow cytometry apoptosis unveiled that HG administration up-regulated the apoptosis of HUVECs, however circVEGFC knockdown decreased the apoptosis (Figure 2E). Ethynyl-2-deoxyuridine (EdU) demonstrated that HG administration repressed the proliferation of HUVECs and circVEGFC knockdown recovered it (Figure 2F). Collectively, HG administration accelerated the apoptosis of HUVECs, and circVEGFC knockdown alleviated it.

### circVEGFC sponged miR-338-3p in HUVECs

**Figure 3:**
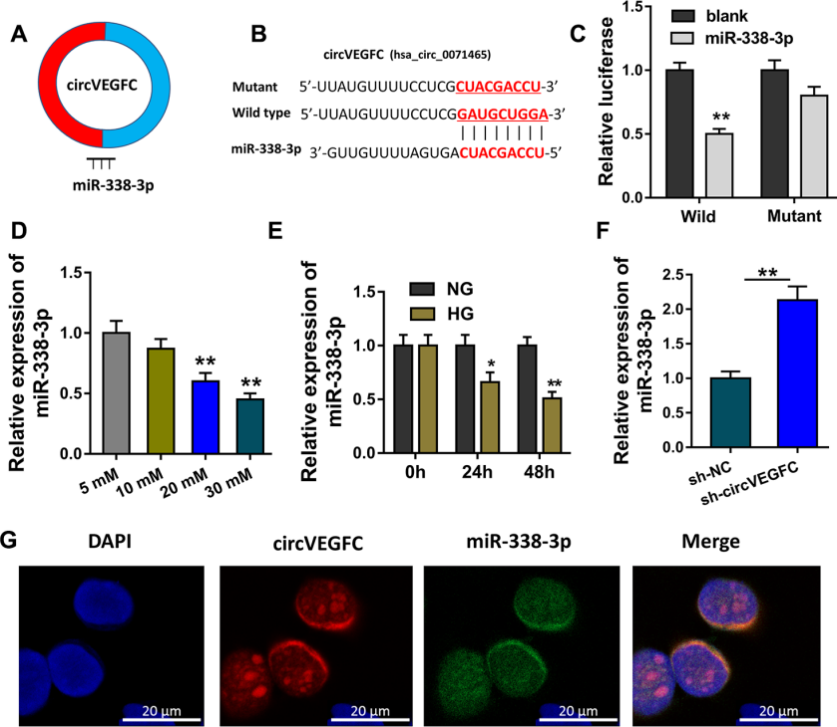
**circVEGFC sponged miR-338-3p in HUVECs.** (**A**) Schematic diagram showed the binding within miR-338-3p and circVEGFC. (**B**) Bioinformatics prediction (https://circinteractome.nia.nih.gov/) indicated the complementary binding sites with circVEGFC. (**C**) Luciferase reporter assay uncovered the compact binding within miR-338-3p and wild type of circVEGFC in the co-transfection. (**D**) RT-qPCR revealed miR-338-3p leve with the concentration rising. (**E**) RT-qPCR revealed the miR-338-3p level in the concentration gradient treated HUVECs. (**F**) RT-PCR showed the miR-338-3p level when circVEGFC was silenced. (**G**) RNA Fluorescence in situ hybridization (RNA-FISH) unveiled the subcellular location of miR-338-3p and circVEGFC in HUVECs. *P < 0.05, **P < 0.01.

Because circVEGFC was generated from the exon of VEGFC, we assumed that circVEGFC might located in the cytoplasmic distribution and function as the sponge of miRNAs (Figure 3A). Bioinformatics prediction (https://circinteractome.nia.nih.gov/) indicated that miR-338-3p shared the complementary binding sites with circVEGFC (Figure 3B). Luciferase reporter assay uncovered that miR-338-3p compactly bound with wild type of circVEGFC in the co-transfection (Figure 3C). In the concentration gradient treated HUVECs, miR-338-3p level was decreased with the concentration rising (Figure 3D). Moreover, miR-338-3p level was also decreased with the time rising (Figure 3E). RT-PCR showed that miR-338-3p was increased when circVEGFC was silenced (Figure 3F). RNA Fluorescence in situ hybridization (RNA-FISH) unveiled that miR-338-3p and circVEGFC were mainly located in the cytoplasmic portion of HUVECs (Figure 3G). Collectively, these data unveiled that circVEGFC sponged miR-338-3p in HUVECs.

### HIF-1α was found to be the target of circVEGFC/miR-338-3p

**Figure 4:**
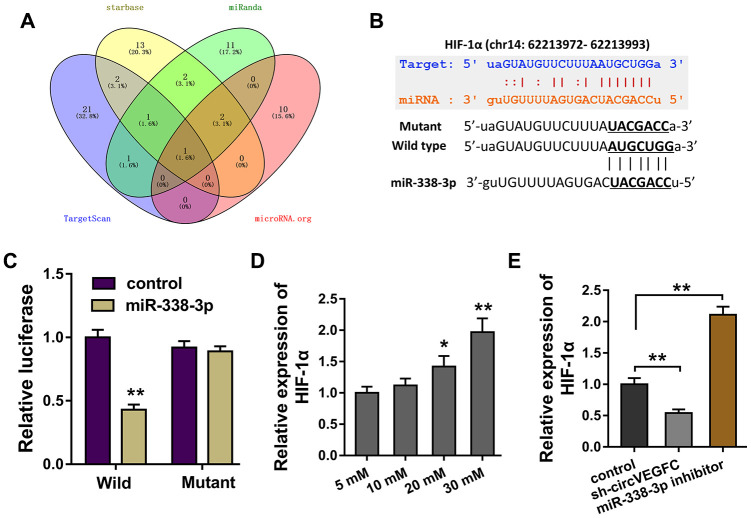
**circVEGFC/miR-338-3p targeted HIF-1α.** (**A**) Bioinformatics tools demonstrated the potential target for circVEGFC/miR-338-3p regulation. (**B**) Wild type and mutant sequence vectors that containing miR-338-3p binding sites were constructed for luciferase reporter assay. (**C**) Luciferase reporter assay illustrated the luciferase activity in the co-transfection of miR-338-3p and HIF-1α wild type or mutant. (**D**) RT-PCR showed the HIF-1α mRNA level in the HG-induced HUVECs. (**E**) RT-PCR showed the HIF-1α mRNA expression in HUVECs transfected with circVEGFC knockdown or miR-338-3p inhibitor. *P < 0.05, **P < 0.01.

Bioinformatics tools demonstrated that HIF-1α was found to be the potential target for circVEGFC/miR-338-3p regulation (Figure 4A). Wild type and mutant sequence vectors that containing miR-338-3p binding sites were constructed for luciferase reporter assay (Figure 4B). Luciferase reporter assay illustrated that miR-338-3p closely conjugated with the wild type of HIF-1α, rather than the mutant plasmids (Figure 4C). In the HG-induced HUVECs, the level of HIF-1α was found to be up-regulated (Figure 4D). Interestingly, HIF-1α mRNA was decreased in circVEGFC knockdown transfection. Besides, HIF-1α mRNA was increased in the miR-338-3p inhibitor transfection (Figure 4E). Therefore, we could find that HIF-1α was the target of circVEGFC/miR-338-3p axis.

### HIF-1α activated the VEGFA transcriptional level in HUVECs

**Figure 5:**
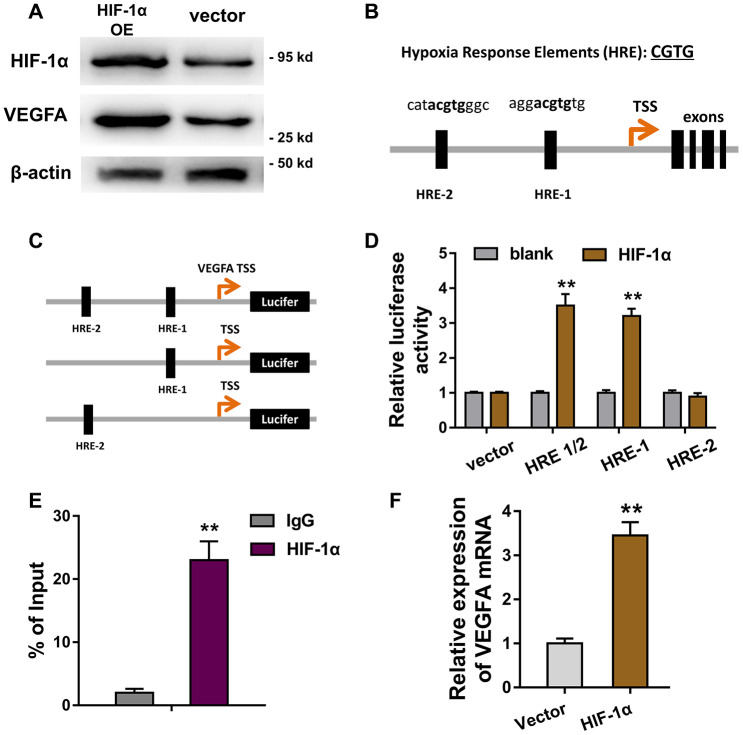
**HIF-1α activated the VEGFA transcriptional level in HUVECs.** (**A**) Western blot revealed the VEGFA protein expression in HUVECs transfected with HIF-1α overexpression plasmids (OE) or controls. (**B**) Bioinformatics analysis illustrated that there were two hypoxia response elements (HRE) in the promoter region of VEGFA gene. (**C**) The plasmid vectors containing HRE-1 and/or HRE-2 were constructed for luciferase assay. (**D**) Luciferase assay showed the Luciferase activity in the transfection with HRE-1 or HRE-2 vectors and HIF-1α. (**E**) Chromatin immunoprecipitation (ChIP) and qPCR showed the abundance of HRE-1 region in HIF-1α antibody immunoprecipitation. (**F**) RT-PCR showed the VEGFA mRNA level in HUVECs with HIF-1α overexpression or controls. **P < 0.01.

Furthermore, in order to investigate the role of HIF-1α in the ECs’ apoptosis, we discovered the potential target of transcription factor HIF-1α. Results of western blot revealed that HIF-1α overexpression plasmids (OE) accelerated the VEGFA protein expression in HUVECs (Figure 5A). Bioinformatics analysis illustrated that there were two hypoxia response elements (HRE) in the promoter region of VEGFA gene (Figure 5B). Then, the plasmid vectors containing HRE-1 and/or HRE-2 were constructed for luciferase assay (Figure 5C). The results of luciferase assay showed that the vectors containing HRE-1 region displayed high molecular interaction of HIF-1α and VEGFA, indicating the direct binding of HIF-1α towards within VEGFA promoter (Figure 5D). Chromatin immunoprecipitation (ChIP) and qPCR showed that the abundance of HRE-1 region was enriched after the HIF-1α antibody immunoprecipitation (Figure 5E). Subsequently, RT-PCR showed that HIF-1α overexpression might promote the VEGFA mRNA level in HUVECs (Figure 5F). Overall, these finding concluded that HIF-1α activated the VEGFA transcriptional level in HUVECs.

## DISCUSSION

Increasing evidence demonstrate the crucial role of circular RNA (circRNA) in the human pathophysiology [10, 11]. Noticeably, the regulation of circRNA in DM and its complication induced by the high glucose (HG) has been unveiled [12, 13]. The vascular complications of DM are the critical causes of morbidity and mortality among people with diabetes [14]. One of the major pathological characteristics is the endothelial dysfunction induced by HG [15]. The damage of vascular endothelial cell (ECs) always functions as the aggravating factor for diabetic vascular complications.

In the vascular microenvironment of DM patients, the stimulation of HG might trigger the dysregulation of noncoding RNA [16, 17]. Among these noncoding RNA, circRNA is a group of novel hotspot for the epigenetic regulation [18]. In present research, the HG-induced HUVECs were cultured to simulate the ECs under DM pathological status. In the microarray analysis, results revealed the expression of circVEGFC was up-regulated in the HG-induced HUVECs. After that, further research found that circVEGFC had the canonical circular loop structure. Besides, circVEGFC could resist the digestion of RNase. Collectively, these data revealed that circVEGFC is a classical circular transcript in the HUVECs.

Numerous studies have revealed the crucial roles of circRNA in the diabetes mellitus or cardiovascular disease. For example, circular HIPK3 expression is significantly upregulated in diabetic retinas and retinal endothelial cells, and the silencing or overexpressing of circHIPK3 regulates the retinal endothelial cell viability, proliferation, migration, and tube formation in vitro [19]. In hypoxia-induced HUVECs, circRNA microarray analysis found the up-regulated circRNA hsa_circ_0010729 and revealed its regulation through miR-186/HIF-1α axis [20]. In I/R induced HUVECs, circDLGAP4 regulates the cell viability, apoptosis and migration, providing insight as to the molecular mechanism of I/R-induced HECTD1 in endothelial cell dysfunction [21].

Existing evidence has indicated the close interaction of hyperglycemia and hypoxia in diabetes microenvironment and their lesion to ECs [22]. Hyperglycemia has been identified to regulate the ECs’ proliferation and apoptosis in diabetes [23]. Moreover, HIF-1α is a critical element in the hypoxia environment, both diabetes and tumor vessels, to modulate angiogenesis and hypoxia response [24]. In this research, we found that HIF-1α acted as the target of circVEGFC and miR-338-3p, constructing the circVEGFC/miR-338-3p/HIF-1α axis. These finding inspired us that hyperglycemia might have close connection with hypoxic response. Subsequently, we also found that HIF-1α, a critical transcription factor, target the VEGFA genomic promoter region, being implicated in the angiogenesis stimulating factor. For instance, HIF-1α targets its downstream genes glucose transporter-1 and vascular endothelial growth factor A and induced their mRNA and protein expressions [25].

**Figure 6:**
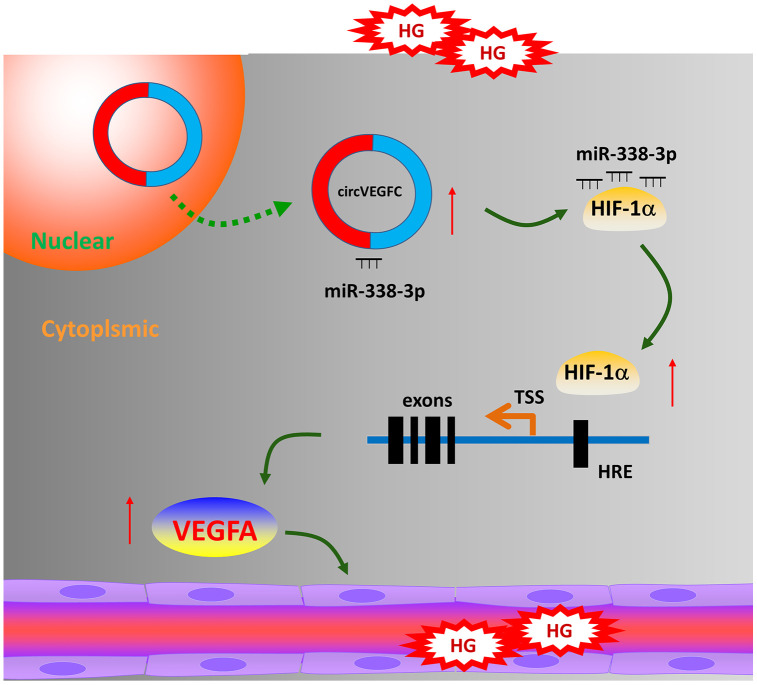
**circVEGFC/miR-338-3p/HIF-1α/VEGFA accelerates the apoptosis in HG-induced HUVECs.**

Collectively, in present research, we reported a novel circRNA circVEGFC in the HG-induced HUVECs and investigated its regulation mechanism for ECs’ apoptosis mediated by HG (Figure 6). circVEGFC accelerates the HG-induced injury by miR-338-3p/HIF-1α/VEGFA axis. These data provides a potential treatment strategy for ECs’ damage in DM.

## MATERIALS AND METHODS

### Cells culture

Human umbilical vein endothelial cells (HUVECs) were purchased from Type Culture Collection of the Chinese Academy of Sciences (Shanghai, China) and cultured in the DMEM/F12 (Dulbecco's modified Eagle medium) as previously described [26]. Medium was supplemented with the 10% fetal bovine serum (FBS, Gibco, Gran Island, NY, USA), endothelial cell growth supplement (ECGS) (BD Biosciences, Bedford, MA) at 37°C and 95% humidity in 5% CO_2_.

### Sanger sequencing and microarray analysis

The PCR products were extracted from the HUVECs, and the Sanger sequencing was performed (Sangon Biotech, Shanghai, China) to detect the full sequence RNA. CircRNA microarray analysis was performed (Arraystar Super RNA Labeling Kit; Arraystar).

### Vector construction and transfection

The methods for vector construction were reported previously. For the overexpression of circVEGFC circRNAs, human circVEGFC cDNA was amplified and inserted into the pCD5-ciR vector (Greenseed, Guangzhou, China). For circVEGFC silencing, miRNA inhibitor and controls were designed and synthesized by Genepharm (Shanghai, China). Transfection was performed using Lipofectamine 2000 (Invitrogen) according to the manufacturer’s instructions. Sequences were listed in Supplementary Table 1.

### RNase R resistance and actinomycin D analysis

The RNA was extracted from HUVECc and then treated with RNase R (4 U/mg) and incubated at 37 °C for 30 min. Then, the level of circVEGFC was detected by qRT-PCR assay with specific primers. Besides, after treatment with Actinomycin D, the level of circVEGFC was detected by qRT-PCR.

### qRT-PCR

Total RNA was extracted using Trizol reagent (Invitrogen) from HUVECs. The cDNA synthesis was performed with Oligo (dT) and the PCR transcript quantification was conducted using specific random primers. Expression levels were quantified using the 2-ΔΔCt method with GAPDH as an internal control. The primers were provided in Supplementary Table 1.

### CCK-8 assay and 5-Ethynyl-2’-deoxyuridine (EdU) assay

HUVECs were seeded in ninety-six well plates (2×10^3^ cells per well) with PBS and 0.25% trypsin and cultured in a 5% CO_2_ incubation at 37 °C. After 24 h incubation, cell counting kit-8 (CCK-8) reagent (Dojindo Laboratories, Kumamoto, Japan) was added and the optical density (OD) value of wells was detected at 450 nm using an automatic enzyme-mark reader. EdU assay kit (RiboBio, China) solution (25 μM) was performed to detect DNA synthesis and cell proliferation. 0.5% TritonX-100 was added. Hoechst 33342 was added to stain the nuclei. DNA synthesis and cell proliferation were observed by Nikon microscope (Nikon, Japan).

### Western blot analysis

HUVECs were lysed and subjected to western blot analysis as described. Total protein form HUVECs was collected by lysis buffer and then separated by 10% SDS-PAGE gel. The isolation was transferred onto the PVDF membrane. The membranes were incubated with primary and secondary antibodies. Antibodies used in the blot were listed as follows: anti-HIF1α (Abcam, ab51608, 1:1000), anti-VEGFA (Abcam, ab52917, 1:1000). The blot signal detection was analyzed using chemiluminescent reagent.

### Flow cytometry apoptosis analysis

HUVECs apoptosis was measured using Annexin V/ Dead Cell Apoptosis Kit (Invitrogen USA). HUVECs were seeded in 6-well plates. After 24 hours starvation, cells were cultured in FBS-free medium. Then, HUVECs were harvested and washed twice with ice-cold PBS. Annexin-binding buffer and 5 μl Annexin V-FITC and 1 μl PI were added to HUVECs for the incubation in the dark. FITC and PI fluorescence were analyzed by flow cytometry (BD Biosciences).

### Luciferase reporter assay

circVEGFC wild type and mutant with potential miR-338-3p binding sites were generated. Sequences were cloned into luciferase reporter vector psi-CHECK-2 (Promega, Madison, WI, USA). Luciferase vectors were conducted using a Dual-Luciferase Reporter Assay System (Promega, USA). HEK293T cells were co-transfected with luciferase plasmids and miR-338-3p or control miRNA. For the luciferase promoter assay, VEGFA promoter wild-type or mutant of the HIF-1α binding was co-transfected into 293K cells using the Lipofectamine 2000 transfection reagent. After 48 h transfection, Renilla/firefly luciferase activities were measured using Dual-Luciferase Reporter Assay System (Promega).

### Fluorescence in situ hybridization (FISH)

In situ hybridization, Cy3-labeled probe for circVEGFC and FAM-labeled miR-338-3p probe were obtained from Genepharma (Shanghai, China). RNA-FISH was performed using fluorescent in situ hybridization kit according to the manufacturer’s protocol (Genepharma). Cell nuclei were stained with 4,6-diamidino-2-phenylindole (DAPI). All procedures were carried out according to the manufacturer’s instructions under a microscope (Olympus, Biological Microscope).

### Chromatin immunoprecipitation

Chromatin immunoprecipitation (ChIP) experiments were performed using Magna ChIP Chromatin Immunoprecipitation Kit according to the manual (Millipore, Billerica, MA, USA). HUVECs were sonicated to decompose the cross-linked DNA into 200 to 1000 bp fragments. The chromatin fraction was incubated with an anti-HIF-1α monoclonal antibody (1:1000, Abcam) at 4°C overnight. The primers are provided in Supplementary Table 1. Immunoglobulin G acted as a negative control.

### Statistical analysis

Statistical analysis was performed using GraphPad Prism version 7.0 and SPSS version 19.0. Statistical differences were calculated using Student t-test, one-way ANOVA. The correlation analysis was calculated using Pearson's correlation. The prognosis was calculated using Kaplan-Meier analysis. P less than 0.05 was considered significant difference.

## Supplementary Material

Supplementary Table 1
