# Supplementary material for: Circular RNA circVEGFC accelerates high glucose-induced vascular endothelial cells apoptosis through miR-338-3p/HIF-1α/VEGFA axis
Source: Aging (Albany NY). 2020 Jul 17;12(14):14365–75. doi: 10.18632/aging.103478 (PMC7425483; doi:10.18632/aging.103478)
Supplement: Supplementary Table 1 [file aging-12-103478-s001..pdf]

SUPPLEMENTARY TABLE

Supplementary Table 1. Sequences of shRNA and qRT-PCR primers.

	5'-3'
circVEGFC	forward, 5'-TGGAAAATGTGCCTGTGAATGT-3'<br>reverse, 5'- ATGTAATTGGTGGGGCAGGTC-3'
sh-VEGFC-1	5'-ATGCAGGTGTCAGGCAGCGAA-3'
sh-VEGFC-2	5'-CCACCAAACATGCAGGTGTCA-3'
sh-VEGFC-3	5'-CACCAAACATGCAGGTGTCAG-3'
miR-338-3p	forward, 5'-GAACCTCCAATAACCGGCC-3'<br>reverse, 5'-GGCGGAGGAACCCCTCATC-3'
HIF1A	forward, 5'-GAACGTCGAAAAGAAAAGTCTCG-3'<br>reverse, 5'-CCTTATCAAGATGCGAACTCACA-3'
VEGFA	forward, 5'-AGGGCAGAATCATCACGAAGT-3'<br>reverse, 5'-AGGGTCTCGATTGGATGGCA-3'
GAPDH	forward, 5'-GGAGCGAGATCCCTCCAAAAT-3'<br>reverse, 5'-GGCTGTTGTCATACTTCTCATGG-3'
